# Supplementary material for: Correlating Cell Behavior with Tissue Topology in Embryonic Epithelia
Source: PLoS One. 2011 Apr 29;6(4):e18081. doi: 10.1371/journal.pone.0018081 (PMC3084706; doi:10.1371/journal.pone.0018081)
Supplement: File S1 — Further details on data acquisition and analysis, and a more detailed discussion of the reduced area concept. Includes three additional figures. (PDF) [file pone.0018081.s001.pdf]

Correlating cell behavior with tissue topology in embryonic epithelia

Supporting Information

S. A. Sandersius[1], M. Chuai[2], C. J. Weijer[2], and T. J. Newman[1,2]

[1]*Center for Biological Physics, Department of Physics, Arizona State University, Tempe, AZ 85287, USA*

[2]*Division of Cell and Developmental Biology, Wellcome Trust Biocentre, College of Life Sciences, University of Dundee, Dundee DD1 5EH, UK*

Supporting Information

A. Data acquisition and analysis

We collect data of cell neighbor numbers (CNN) from the epiblast of the chick embryo at two different developmental stages: pre-streak formation (stage EGXII [3]), and during streak formation (stage HH2 [4]). Figure S1a shows a micrograph of the stage EGXII epiblast and the white square indicates the section size of the tissue we analyzed at higher magnification for pre-streak (Pre-S). Figure S1b shows a micrograph of the stage HH2 epiblast where the two white squares indicates the section sizes of the tissue we analyzed at higher magnification for a region lateral to the streak (LS), and a region within the streak (WS).

Confocal images were generated and maximum projection Z stack analyzed (Figure 1, main paper). CNN were counted manually for each cell. For the Pre-S, LS, and WS micrographs, each cell was manually labeled with its CNN (Figure S2a,c,e). This was done with Adobe Photoshop and the original files are available for download: sdata_PS.psd, sdata_LS.psd, sdata_WS.psd.

A major problem for image analysis is the substantial number of apparent 4-way junctions. An example of an apparent 4-way junction is shown in Figure S1c; in the figure four cells are labeled (4', 5', 6', 6'). The prime denotes the number of unambiguous neighbors of each cell. If all four cells were in contact with each other, the CNN would be (5, 6, 7, 7). However, we consider only three-way junctions, and thus need to resolve this apparent four-way junction into two three-way junctions, such that only two diagonally opposing cells will pick up an extra neighbor. Thus, there are two possibilities for the CNN: (5-7, 5-6) or (4-6, 6-7), where the dash indicates diagonally opposing pairs of cells. This type of systematic analysis was done for every non-resolvable 4-way junction: of which there were 18 for Pre-S, 34 for LS, 28 for WS, and provides a measure of intrinsic error in computing the CNN histograms. Two histograms were compiled for each of Pre-S, LS, and WS (Figures S2b,d,f): either maximizing (blue) or minimizing (red) the number of 6-neighbored cells. The mean histogram is shown in black in each case.

B. Reduced area: a parameter or a distribution?

In a recent paper by Hocevar and Ziherl (HZ) [2], an energy-based model for geometric order was introduced, and shown to be capable of reproducing a range of histograms found in *Drosophila.* Fundamental to the HZ model is the idea that a given tissue is characterized by an "order parameter", namely the reduced area a. This quantity is assumed to be the same for each cell in the tissue, and is defined as 4π times the area of the cell, divided by the square of the cell perimeter. Thus $a = 1$ for a circle, and will be less that unity for other cross-sectional shapes. HZ find that cells in the *Drosophila* imaginal disk have a range of values of a within the same tissue, but argue that given the standard deviation of values is relatively small, the concept of the order parameter is still useful.

We have measured the reduced area of all cells in the chick Pre-S tissue. Reduced areas were measured manually using ImageJ. Each cell was outlined manually using the Freehand Selection tool then area and perimeter were computed using the Analyze/Measure application. These values were then binned and the resulting histogram is shown in Figure S3. The distribution of values is noticeably broader than that found in HZ. Indeed, we find that the standard deviation is twice that measured in HZ. It is therefore quite difficult to maintain that such a tissue can be characterized by a single order parameter. One can, of course, focus instead on the mean value of the reduced area, which in the case of the chick Pre-S is $\langle a \rangle = 0.74 \pm 0.10$.

---

[1] Gibson M C, Patel A B, Nagpal R, Perrimon N (2006) The emergence of geometric order in proliferating metazoan epithelia. *Nature* **442**:1038-1041.

[2] Hocevar A, Ziherl P (2009) Degenerate polygonal tilings in simple animal tissues. *Phys Rev E* **80**:011904 (7pp).

[3] Eyal-Giladi H, Kochav S (1976) From cleavage to primitive streak formation: a complementary normal table and a new look at the first stages of development of the chick. I. General morphology, *Dev Biol* **49**:321-337.

[4] Hamburger V, Hamilton H L (1951) A series of normal stages in the development of the chick embryo. *J Morphol* **88**:49-92.

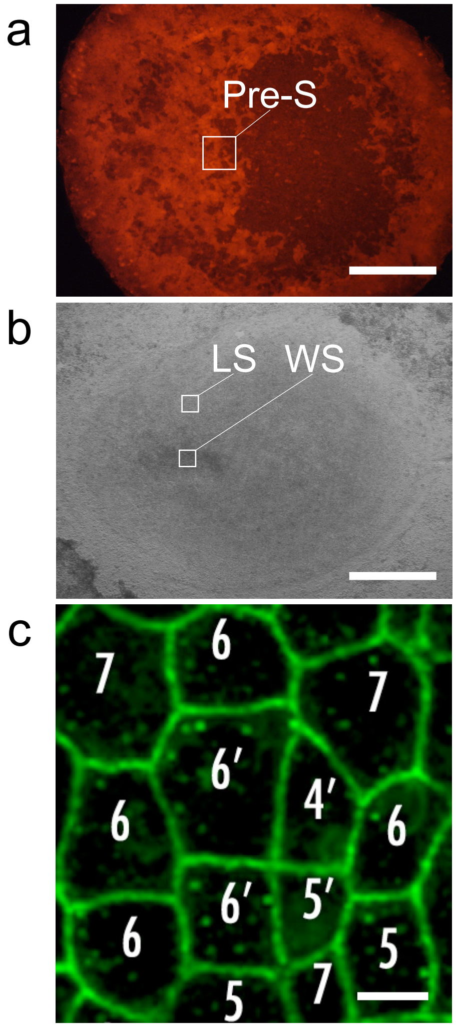

Figure S 1: Early Chick Embryo. (a) Embryo at stage EGXII: pre-streak formation. The white square indicates the size of the tissue sample that was analyzed at higher magnification (Figure S2a) to obtain Pre-S histogram (Figure S2b). (b) Embryo at stage HH2: during streak formation. White squares indicate the size of the tissue samples that were analyzed at higher magnification (Figures S2c,e) to obtain LS, and WS histograms (Figures S2d,f) respectively. (c) Example of an apparent 4-way junction. Top-left cell is in contact with bottom-right, or equally likely, bottom-left cell is in contact with top-right cell. CNN are labeled with primes for systematic error analysis. The scale bars represent 1000 μm in (a) and (b). The scale bar in (c) represents 5 μm.

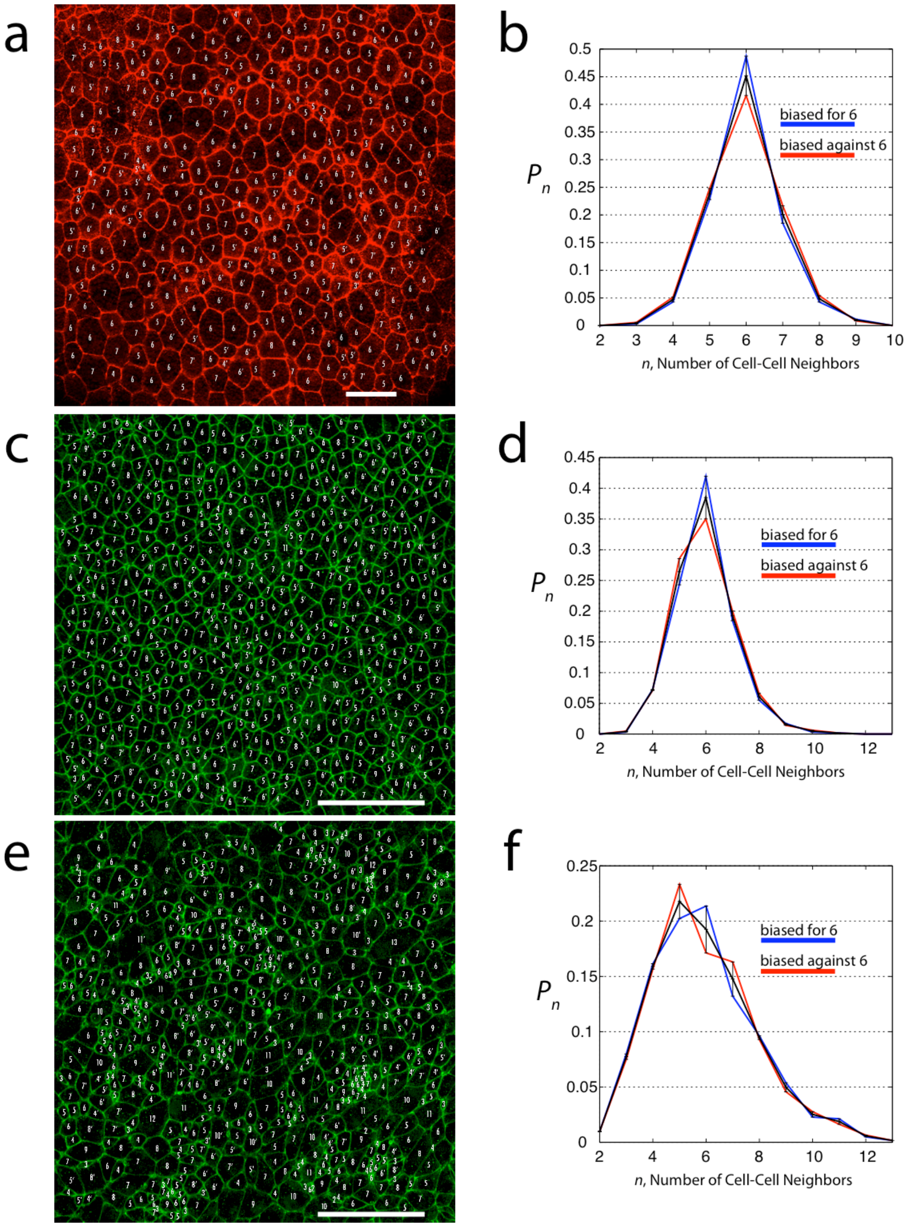

Figure S 2: High magnification images of small areas of the chick embryo showing each cell labeled with its CNN. (a,b) Stage EG XII, prior to streak formation (Pre-S); (c,d) stage HH3, lateral to the streak (LS); (e,f): stage HH2, within the streak region (WS). The embryo in image (a) is stained with rhodamine phalloidin to visualize the F actin cortex, while the embryos shown in (b) and (c) are stained with an antibody against the apically localized tight junction marker ZO-1. The scale bars represent 50 μm in (a), (c), and (e). Panels (b), (d), and (f) show CNN histograms for (a), (c), and (e) respectively.

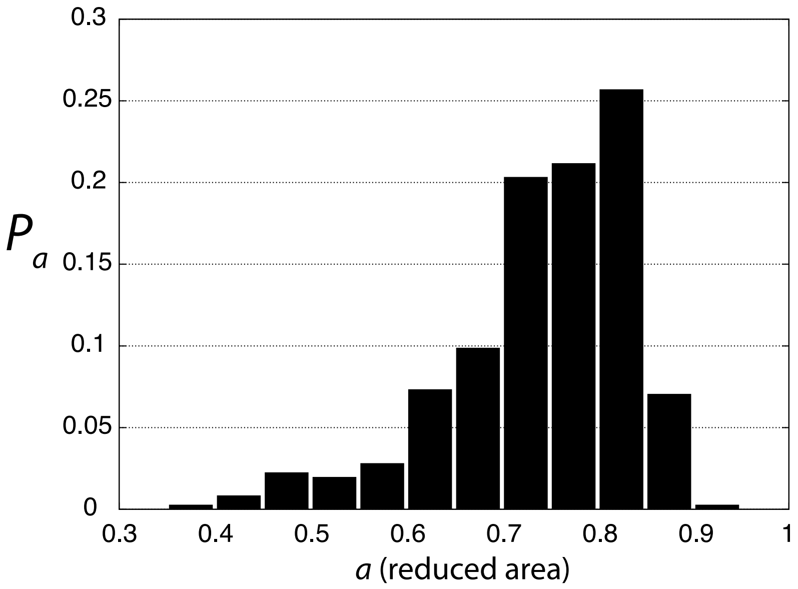

Figure S 3: Histogram of reduced area measured for 354 cells shown in Pre-S (Figures 1a, and S2a). The histogram shows a broad distribution of reduced area.
